# Landslide Susceptibility Evaluation Integrating Machine Learning and SBAS-InSAR-Derived Deformation Characteristics: A Case Study of Yining County, Xinjiang

**DOI:** 10.3390/s26020707

**Published:** 2026-01-21

**Authors:** Tingting Ma, Xiaoqiang Yi, Hui Ci, Ran Wang, Hui Yang, Zhaojin Yan

**Affiliations:** 1 Department of Energy and Chemical Engineering, Aksu Industry Polytechnic College, Aksu 843100, China; ts22010135p31@cumt.edu.cn; 2 School of Resources and Geosciences, China University of Mining and Technology, Xuzhou 221116, China

**Keywords:** landslide disasters, Yining County, machine learning, SBAS-InSAR, landslide susceptibility assessment

## Abstract

Against the background of intensified climate change and enhanced human activities, the occurrence mode of landslides is becoming more complex and changeable, showing a trend of clustering, contiguous, and frequent occurrences. Yining County is located in the middle of the Yili River Valley, where the geological conditions are fragile, neotectonic movement is active, and landslide disasters are widely developed and frequent, posing a serious threat to the population, buildings, and infrastructure. Based on multi-source data combined with machine learning models and SBAS-InSAR technology, this paper realized refined landslide susceptibility evaluation. Firstly, through correlation analysis and other methods, 12 landslide evaluation factors were selected, and the ChiMerge method was used to discretize the continuous factors to build the landslide susceptibility evaluation system. Four machine learning models were used to predict landslide susceptibility, and the RF model performed best. Using the dynamic timeliness advantage of SBAS-InSAR technology, the optimized regional landslide susceptibility evaluation results were constructed, which improved the precision of the landslide susceptibility evaluation results. The purpose of this study is to improve the accuracy and timeliness of landslide sensitivity assessment, improve regional disaster prevention and emergency management planning ability, and provide theoretical and data support for local sustainable development.

## 1. Introduction

A landslide is a geological phenomenon triggered by the combined action of internal and external factors such as geological structures and rainfall, which causes the rock and soil mass of the slope to produce shear displacement and slide downward along the weak structural plane of the slope [1]. It is a major global disaster that has the characteristics of strong suddenness, great harm, and high difficulty in treatment [2]. Heavy rainfall [3], snow melting [4], earthquakes [5], volcanic eruptions [6], climate change [7], land cover changes [8], and human activities [9] may induce landslides [10,11].

Landslide disasters mainly occur in mountainous and hilly areas with steep terrain and complex terrain [12]. China is one of the countries that has experienced severe casualties from landslides, owing to its complex and varied topographical features and the frequent occurrences of landslide disasters [13,14,15]. Yining County is located in the Yili River Valley in central and western Xinjiang. The geological environment is complex and changeable, the overall terrain fluctuates greatly, and neotectonic movement is active. Combined with seasonal precipitation concentration and ice and snow freezing and thawing, landslide disasters have become the most developed geological disaster type in Yining County, posing a serious threat to people’s lives and property. Therefore, it is particularly important for urban construction planning, geological disaster prevention, and regional sustainable development to monitor landslide disasters in a timely manner and carry out susceptibility assessment and mapping.

Landslide susceptibility assessment refers to the quantitative assessment and pre-diction of the possible location and probability of landslide disasters, as future landslides are likely to occur under conditions similar to historical landslide condition factors, and the landslide condition factors that affect the occurrence of landslides are spatially related [16,17]. Therefore, landslide susceptibility analysis can be realized through historical landslide catalog data and relevant geospatial landslide factor data [18].

At present, landslide susceptibility models are generally grouped into three families: heuristic [19], statistical [20], and machine learning [21]. Because heuristic methods are inherently subjective and statistical models struggle to capture nonlinear relationships, machine learning as a typical data-driven tool has become the mainstream method for susceptibility mapping [22]. Among the machine learning methods, Logistic Regression (LR) [23], Support Vector Machine (SVM) [24], Random Forest (RF) [25], and Artificial Neural Network (ANN) [26] are the most frequently adopted. Their strong fitting capacity and high prediction accuracy have attracted extensive attention from researchers worldwide. Ali et al. used RF and the feature selection method to identify landslide parameters and improve the accuracy of landslide susceptibility mapping [27]. M. Youssef et al. coupled machine learning with spatial data to predict landslide susceptibility and evaluated the accuracy and performance of seven kinds of machine learning models in landslide susceptibility modeling [28]. Xiao et al. combined RF and SHAP, achieved the optimal landslide susceptibility assessment, and gave an interpretation of Northwest Yunnan Province, China [29].

Using machine learning methods can effectively fit the nonlinear relationship be-tween environmental factors and landslide susceptibility factors and can reduce the uncertainty level of model prediction by analyzing the complex dataset of landslide susceptibility. Moreover, machine learning has good robustness in noisy environments and does not need special rule statistics [30]. Because different models differ in adapting to different geological environments, data characteristics, and landslide occurrence mechanisms, there is no universal machine learning model suitable for landslide susceptibility evaluation in all regions [31]. Zhang et al. used RF, XGBboost, and other methods to evaluate the landslides in the Henan section of the Yellow River Basin, and found that under complex geological and rainfall conditions, RF (AUC = 0.9599) performed best [32]. Shen et al. proposed a comprehensive landslide susceptibility model based on the combination of the information value model and integrated machine learning model. The prediction effect of IVW combined with the XGBoost model was the best [33]. Therefore, in specific research, it is usually necessary to compare and evaluate a variety of machine learning models and select models suitable for the characteristics of the study area to improve the prediction accuracy and reliability of landslide susceptibility assessment [34].

However, since the machine learning model is built by analyzing the relationship between the distribution of historical landslides and environmental factors, its prediction accuracy depends on a key premise: the occurrence conditions of future landslides are similar to those of historical landslides [35]. Under the background of significant climate change and land use change, human activities are prone to induce landslide events with new characteristics. The most direct external manifestation of landslide movement is land deformation, and the deformation rate and variables can reflect the evolution stage of the landslide mass. SBAS-InSAR technology can realize regional-scale land deformation monitoring and mapping [36], so as to introduce near-real-time landslide deformation indicators for susceptibility evaluation [37,38,39]. Zhou et al. combined MT-InSAR and machine learning models to propose a new physical-based, cost-effective landslide displacement prediction framework, which could effectively predict landslide displacement in a large range [40]. Hussain et al. combined the PS-InSAR results with the landslide susceptibility results generated by the XGBoost to create a new landslide susceptibility map for the areas along the Karakoram Highway [41]. Yan et al. drew a dynamic landslide hazard map of Zigui County in the Three Gorges Reservoir area from 2019 to 2021 by combining InSAR deformation rate zoning and RF model landslide susceptibility results [42]. Wei et al. used a machine learning model based on RF, LR, and XGBoost with InSAR technology. It was confirmed that the combined method improved the prediction accuracy of dynamic landslide susceptibility and effectively reduced the false-negative and false-positive errors [43]. Zhou et al. found that InSAR technology effectively corrected the overestimation and underestimation of landslide susceptibility in the catalog map and improved the accuracy and timeliness of landslide susceptibility mapping [44]. Li et al. developed a high-precision coupling method of Random Forest and slope enhancement to improve the accuracy of landslide prediction [45]. Therefore, by monitoring the land deformation characteristics of landslides and integrating the deformation results into machine learning, it is beneficial for improving the accuracy of landslide susceptibility assessment.

This study established an integrated assessment framework that combines machine learning-based landslide susceptibility evaluation with SBAS-InSAR surface deformation data. By introducing a dynamic post-processing correction mechanism based on deformation information, a deep coupling between near-real-time deformation features and regional landslide susceptibility analysis was achieved. While ensuring the structural stability of the machine learning model, this method significantly enhanced the timeliness and geospatial consistency of susceptibility zoning results, effectively addressing the common issues of spatial overestimation or underestimation in traditional evaluation methods. Validated in the typical complex mountainous area of Yining County, Xinjiang, this framework provides a valuable technical reference for landslide monitoring and risk assessment in similar mountainous regions.

## 2. Materials and Methods

### 2.1. Study Area

**Figure 1:**
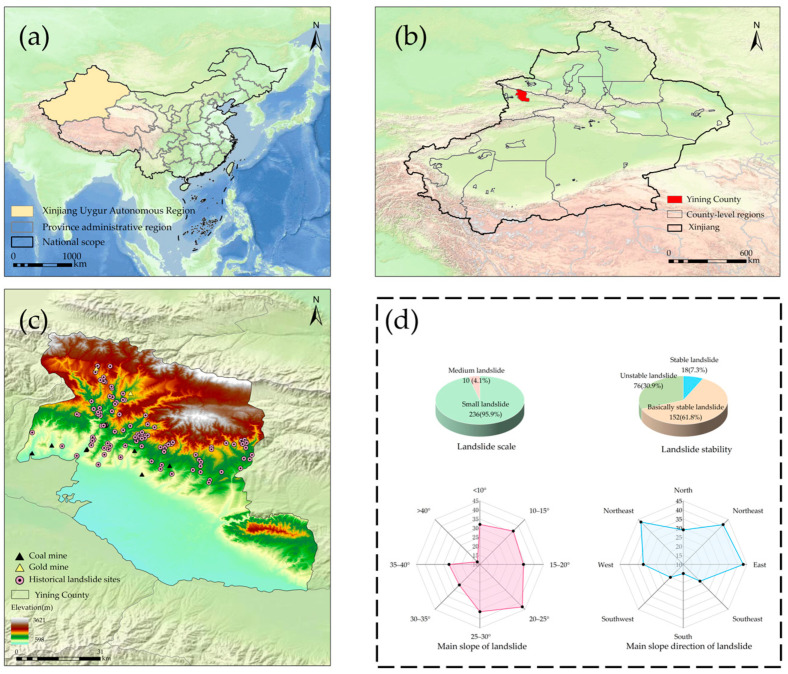
Overview of study area: (**a**) the location of Xinjiang Province; (**b**) the location of Yining County in Xinjiang Province; (**c**) overview of Yining County; (**d**) classification of landslide.

Yining County (43°35′10″~44°29′30″ N, 81°13′40″~82°42′20″ E) is located in the west of Xinjiang, the western edge of the northern slope of the Tianshan Mountains, and the north central part of the Yili River Valley (Figure 1a,b), with a total area of about 6152 km^2^. The climate is a temperate continental semiarid climate, with four distinct seasons: warm and humid winter and spring and dry and hot summer and autumn. Due to the influence of special terrain, the territory is rich in precipitation and water resources. The county has an average annual precipitation of 340 mm, with surface water runoff reaching 4.346 billion cubic meters and groundwater recharge totaling 908 million cubic meters, of which 573 million cubic meters are exploitable. In summer, influenced by westerly moisture and localized convective weather, precipitation is concentrated and often characterized by heavy rainfall. Short-duration intense rainfall can rapidly infiltrate mountain rock and soil layers, making this period the most prone to landslides.

The overall terrain is high in the north and low in the South (Figure 1c) and inclined from northeast to southwest, and the landform types are complex and diverse. From north to south, it can be divided into mountains, hills (platforms), and plains. The mountainous area accounts for more than 70% of the total area, mainly focusing on animal husbandry, forestry, and mining. Frequent human engineering activities such as transportation facility construction and tourism development have caused varying degrees of changes and even damage to the geological environment, resulting in mountain disturbance and increasing landslide risk. In the process of mining, mountains are usually excavated in different forms, especially explosive blasting, which seriously affects the stability of the mountains and destroys the stress distribution in the mountains. Moreover, the mining waste rock is dumped in the valley, which increases the slope load and provides material source conditions for landslides. During the construction of the highway, a large number of steep cut slopes of artificial mountains were formed, resulting in a large free surface on the slope. When artificial slope cutting occurs on the bedding slope or when there is water flowing into the seepage, the risk of slope instability increases, which may lead to rockfall, landslide and slope collapse, and debris flow.

Summer is the main flood season in Yining County. Influenced by westerly moisture and local convective weather, rainfall exhibits pronounced concentration and short-duration high-intensity characteristics, with daily precipitation in some mountainous areas reaching 50–80 mm. Statistics indicate that approximately 60–70% of landslide events in the county occur during summer, with mostly shallow landslides being triggered by short-term heavy rainfall, and their spatiotemporal distribution closely aligns with intense rainstorm events. By the end of 2020, a total of 235 potential geological hazard sites had been identified in the county, of which 218 were landslides, directly threatening 503 residents and posing a potential property loss of about CNY 15.1 million (https://www.xjyn.gov.cn/xjyn/c113700/202311/2f9ae7f08ddf4889af9543b769b0ce99.shtml, accessed on 1 January 2025). On this basis, through remote-sensing interpretation and field verification, this study systematically supplements and verifies regional landslide records, integrating a total of 246 landslide events (Figure 1d). Most of these landslides are small-scale, with no large-scale landslides. In terms of stability, most landslides are basically stable, followed in frequency by unstable ones. The distribution of landslides is mainly within a slope gradient of 40°, among which the largest number of landslides occur at 20~25°. There is a linear positive correlation between terrain slope gradient and stress; the slope stress within this range is conducive to landslide occurrence, and landslides are more likely to be triggered under the influence of external factors such as rainfall and snowmelt. When the slope gradient exceeds 40°, concentrated shear stress tends to form inside the slope surface, making collapse-slide-type landslides prone to occurring. After the sliding mass material becomes unstable, it quickly detaches along the sliding bed and reaches a new state of equilibrium rapidly. Landslides are distributed across all slope aspects, but predominantly occur on north-facing slopes. North-facing slopes are shady slopes, where soil water content is higher than that of sunny slopes. Under rainfall condition, the soil is more likely to reach a saturated state, which leads to a significant reduction in the shear strength on structural planes in the soil or at rock–soil interfaces, thereby inducing landslides.

However, extreme rainfall events (high-intensity rainfall exceeding 40 mm per day in Yining County) and human activities have exacerbated the threat of soil erosion and geological disasters and have weakened progress in achieving the goal of sustainable development. Conducting a susceptibility assessment of Yining County is very important for formulating strategies that take into account disaster mitigation, ecological protection, and resilient development, which is also in line with the goal of regional sustainable growth.

### 2.2. Data

#### 2.2.1. Landslide Driving Factor Data

**Figure 2:**
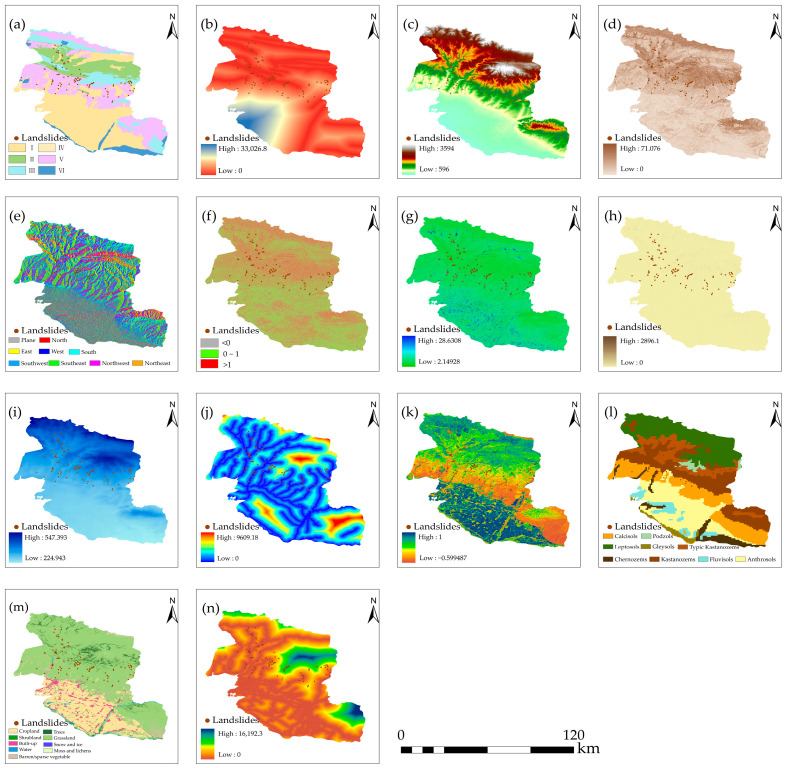
Landslide evaluation system: (**a**) lithology (I: single-layered loess-like sandy loam soil mass; II: layered, relatively hard, volcaniclastic rock formation; III: massive hard granite and diorite rock formation; IV: multi-layered soil mass containing boulders, cobbles, and sandy loam; V: interbedded, relatively weak formation dominated by sandstone and conglomerate; VI: single-layered gravel and sand soil mass.); (**b**) Distance from Fault; (**c**) DEM; (**d**) slope; (**e**) aspect; (**f**) profile curvature; (**g**) Topographic Wetness Index (TWI); (**h**) Sediment Transport Index (STI); (**i**) Annual Average Precipitation; (**j**) Distance from River; (**k**) NDVI; (**l**) soil type; (**m**) land use; (**n**) Distance from Road.

Based on an extensive analysis of the literature combined with the geological environmental characteristics of landslide occurrence in the study area, this paper collected 14 factors from landslide driving factor data, including engineering geology, topographic features, hydrometeor, land cover, and human activity (Figure 2) [46,47,48,49]. Among these, the lithology data were obtained by converting the original vector format into raster data, while the Distance from Fault, the Distance from River, and the Distance from Road were generated by calculating the Euclidean distance based on vector lines. All data were resampled to a uniform spatial resolution of 30 m × 30 m and projected to the WGS_1984_UTM_Zone_44N coordinate system. Relevant information about the data is shown in Table 1.

Rock and soil mass are the material basis of a landslide. The structure and mechanical properties of different types of rock and soil mass are different, which is an important factor to determine whether an area is landslide-prone [50]. Faults have a great impact on the displacement and deformation of slopes, and the strong fracture zone formed at the fault crossing is the basis for the occurrence of loose-accumulation landslides [51].

Elevation is one of basic influencing factors of landslides, which directly affects the stress on the slope and the regional climate conditions, leading to the difference in slope stability [52]. Slope affects the shear stress of a landslide mass [53]. With the increase in slope, the shear stress also increases. When the shear stress reaches or exceeds the shear strength of the slope material, landslides may occur. Aspect controls climate parameters such as soil moisture and has an indirect impact on the occurrence of landslides [54]. The vegetation on the shady slope is often sparse, the soil is wet, the slope is easily saturated during precipitation, and the stability of the slope is reduced, leading to landslide disasters. STI is an index used to describe the movement rate of solid particles and to control the movement process, and can be used to characterize the erosion intensity of surface runoff. The larger the STI value, the thicker the accumulated sediment, and the more prone the area to landslides [55]. TWI is a crucial index for describing the terrain’s capacity to accumulate moisture [48]. It reflects the spatial distribution pattern of soil moisture by quantifying topographic features. Areas with low values are mostly distributed on hillsides or in steep terrain. These regions have relatively large slopes, leading to rapid moisture loss and relatively dry soil. The profile curvature is the second derivative of elevation change, and its value reflects the change rate of a slope [56]. Its value describes the concave and convex nature of the terrain, which can be divided into a concave slope (>1), straight slope (0~1), and convex slope (<0).

Rainfall is one of the driving factors of landslides and can cause slope instability [57]. It increases the water content of the slope, reduces the shear strength of the soil, and easily forms weak structural planes or ground fissures in rock and soil. This promotes slope creep and ultimately leads to landslides. River systems exert scouring and eroding effects on slope toes. They increase the free face of the slope, reduce the stability of rock and soil mass, and thus raise the possibility of landslides [51]. NDVI can represent the vegetation cover status. Vegetation cover generally contributes to slope stability; the water absorption effect of plants keeps slopes with vegetation cover 12.84% drier on average than bare slopes [58]. Meanwhile, vegetation reinforces the soil through its root system to enhance slope stability and intercepts rainwater to reduce the scouring effect of rainfall infiltration on the slope surface. Soil type reflects the texture and composition of the landslide soil, serving as the material basis for landslide formation [59]. Land use is a dynamic process shaped by the combined effects of environmental changes and human activities [60]. Changes in land use affect hydrological processes such as runoff, evapotranspiration, and infiltration. Most roads in the study area are built along riverbank slopes and their toes. Road construction involves activities such as slope cutting and foundation excavation. These activities often alter the natural topography of the slope, increase the slope load, and induce landslide disasters [60].

#### 2.2.2. SAR Image Data

The SAR image data used in this paper are Sentinel-1A Single Look Complex (SLC) images, with VV polarization, Interferometric Wide (IW) imaging mode, and ascending orbit direction [60]. To obtain Sentinel-1A data covering the entire study area, two adjacent images with Path 85 and Frames 143 and 138 were used for mosaicking. The data span from 2 January 2022 to 23 December 2023, with a temporal interval of 12 days, resulting in a total of 118 SAR images.

### 2.3. Method

**Figure 3:**
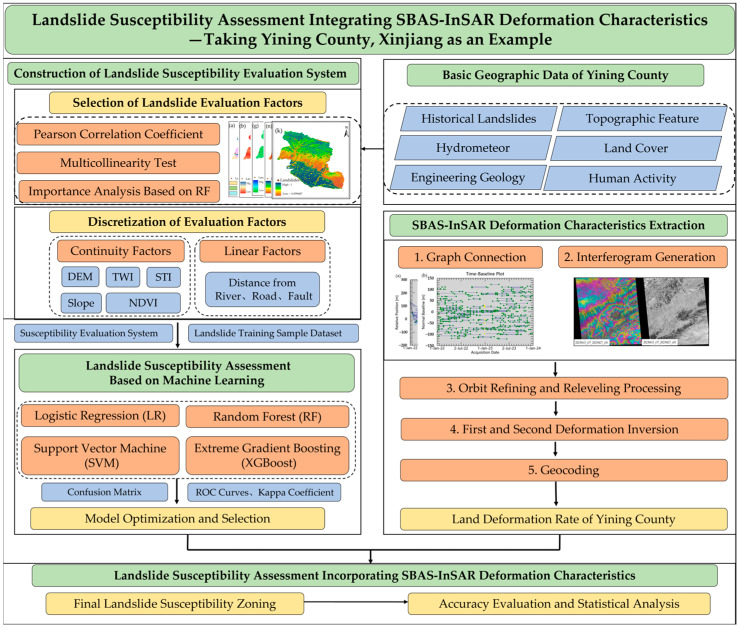
Study process.

The research process of this paper is shown in Figure 3. The overall framework consists of several key steps: first, based on the basic geographic data of Yining County, influencing factors were screened using methods such as the Pearson correlation coefficient, and continuous data were discretized to construct the susceptibility evaluation index system; then, machine learning methods were applied, and evaluation metrics such as the confusion matrix were used to select the model most suitable for the study area; finally, by integrating the surface deformation rate results obtained through SBAS-InSAR technology, the final susceptibility zoning and accuracy assessment were completed.

#### 2.3.1. Selection of Landslide Evaluation Factors

High correlation among landslide evaluation factors can introduce noise and reduce the predictive ability of landslide susceptibility models. Therefore, in this study, three methods—Pearson correlation coefficient, multicollinearity test, and RF importance analysis—were used to screen evaluation indicators, aiming to establish an evaluation indicator system suitable for model training.

First, this paper used the Pearson correlation coefficient to analyze the linear correlation between pairwise variables. The value range of the Pearson correlation coefficient is [−1, 1]; the closer the absolute value is to 1, the stronger the correlation between variables. In this paper, it was set that if the correlation between a factor and other factors $\left|\gamma \right|>0.7$, it was regarded as a redundancy factor [61].

Multicollinearity refers to the phenomenon that the regression estimation is inaccurate due to the high correlation between variables in the multiple regression model. After preliminary screening by bivariate correlation analysis, in order to further reduce the risk of model overfitting and optimize the process of variable selection, we used the Variance Inflation Factor (VIF) and Tolerance Level (TOL) to evaluate the multicollinearity between evaluation factors. When VIF < 10 and TOL > 0.1, it is considered that there is no significant multicollinearity problem between indicators [62].

In order to understand the impact of each evaluation factor on landslide susceptibility, the Gini index in RF was used to score the importance of evaluation factors. This method evaluated the importance of index variables based on the reduction in Gini impurity. In each split, the reduction in node impurity was calculated. The greater the reduction in impurity in the split, the higher the importance of the factor. The average value of Gini impurity reduction in an indicator in all trees was the importance score of the feature.

#### 2.3.2. Discretization of Landslide Evaluation Factors

To enhance the efficiency of model analysis and ensure the precision of predictions, it was essential to discretize the continuous data within the selected evaluation metrics. This paper employed the ChiMerge discretization method to process the continuous data. The ChiMerge algorithm assessed the independence of adjacent intervals using the Chi-square statistic ($\chi^{2}$) and merged intervals based on the guidance of output variables, thereby better reflecting the correlation between data distribution characteristics and target variables. The calculation formula of $\chi^{2}$ is:

(1) $$\chi^{2}=\sum_{i=1}^{2}\sum_{j=1}^{k}\frac{{\left(A_{ij}-E_{ij}\right)}^{2}}{E_{ij}}$$

(2) $$E_{ij}=R_{i}\times C_{j}/N$$

(3) $$R_{i}=\sum_{j=1}^{k}A_{ij}$$

(4) $$C_{j}=\sum_{j=1}^{2}A_{ij}$$

(5) $$N=\sum_{i=1}^{2}R_{i}$$

where k is the number of categories; $A_{ij}$ is the number of j samples in i interval; $E_{ij}$ represents the number of samples of j in interval i according to the proportion of samples of j in the population; $R_{i}$ represents the number of samples in interval i; $C_{j}$ the number of samples of class j; and N is the total number of samples.

#### 2.3.3. Landslide Susceptibility Evaluation Model Based on Machine Learning

To select a more suitable machine learning prediction model for Yining County, we established four models, namely LR, SVM, RF, and XGBoost. Meanwhile, we adopted the Optuna hyperparameter optimization framework and used the Bayesian optimization algorithm to automatically optimize the key parameters of each model to obtain the optimal model parameter combination.

LR is a regression analysis with a dependent variable as the binary classification variable, which aims to explore the relationship between the dependent variable and independent variable and characterize the correlation between them by fitting a linear model. In this paper, L2 regularization and lbfgs solver were selected, and the regularization parameter was determined to be 0.0298 through logarithmic space search. In order to ensure the convergence of the model, the maximum number of iterations was set to 1000.

SVM mainly realizes classification by constructing hyperplane and maps the data from the original space to the high-dimensional feature space, making the originally inseparable data separable in the high-dimensional space. In the process of parameter optimization, the logarithmic space sampling method was used to search the parameters of the kernel function, and finally the optimal parameter combination of the RBF kernel function was determined, in which the C value was 0.255 and the γ value was 8.57 × 10^−5^.

RF generates multiple data subsets by random sampling, builds multiple independent decision trees on different subsets, and finally integrates the prediction results of each decision tree by majority voting, producing more stable and accurate results. We used a uniform integer sampling method to search the number of decision trees and the maximum depth of trees. After 50 iterations, the optimal parameter combination of n_estimators = 347 and max_depth = 15 was selected.

XGBoost builds a strong learner by gradually accumulating multiple weak learners. The training goal of weak learner is to minimize the prediction error of the previous model to optimize the objective function and achieve accurate prediction of the target variables. In this paper, the weighted loss function of L2 regularization (λ=1) was used to control the complexity of the model and prevent overfitting. In terms of parameter optimization, the Bayesian optimization framework was used to systematically optimize the core parameters of XGBoost. Finally, the number of decision trees was 306, the learning rate was 0.0065, and the maximum depth of the tree was 10.

#### 2.3.4. SBAS-InSAR Interferometric Processing

**Figure 4:**
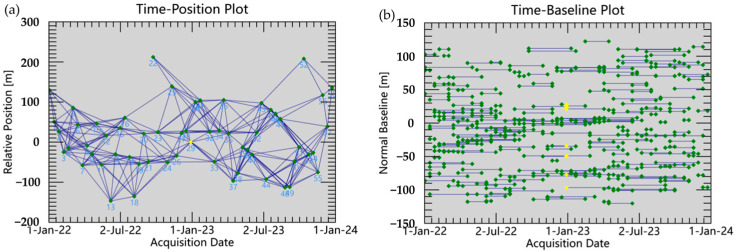
Spatiotemporal baseline connection diagram of image interference pairs. The yellow dot represents the reference master image and the green dot represents the remaining 58 valid SLC images. The line represents the connection between the super master image and the slave image. The figures represent the relative position relationship between the influences in different periods.

We carried out SBAS-InSAR interferometric processing by the SARScape5.7 based on the ENVI5.6 platform, which mainly included the key steps of connection graph generation, differential interferogram generation, phase unwrapping, orbit refining and releveling processing, deformation inversion, and geocoding. Figure 4 shows the temporal and spatial baseline connection diagram relative to the master image. We set the spatial baseline threshold to 2% and the maximum temporal baseline to 120 days for interferometric pairing, and selected the SAR image acquired on 28 December 2022 as the super master image. A total of 280 interferometric pairs were generated, among which each individual image formed at least 3 image pairs and a maximum of 14 image pairs, with an average of 9.49 image pairs.

#### 2.3.5. Landslide Susceptibility Transfer Matrix

The transfer matrix can comprehensively and specifically describe the directional and structural characteristics of changes among various landslide susceptibility types, while also clearly reflecting the loss and transfer relationships between the initial susceptibility zoning results and the susceptibility zoning results integrated with SBAS-InSAR deformation data. Its mathematical expression is as follows:

(6) $$S_{ij}=\left|\begin{array}{cc} \begin{array}{cc} S_{11} & S_{12}\\ S_{21} & S_{22} \end{array} & \begin{array}{cc} \ldots & S_{1n}\\ \ldots & S_{2n} \end{array}\\ \begin{array}{cc} \vdots & \vdots \\ S_{n1} & S_{n2} \end{array} & \begin{array}{cc} \vdots & \vdots \\ \ldots & S_{nn} \end{array} \end{array}\right|$$

where n is the total number of susceptibility categories involved in the transition; S is the area of each type; i and j are the pre-transition and post-transition susceptibility types, respectively; and $S_{ij}$ is the area transitioning from type i before the transition to type j after the transition. The row elements of this matrix reflect the outflow structure of an area from a given pre-transition type to all other types, while the column elements correspond to the inflow sources of an area for a given post-transition type from all pre-transition types, thereby systematically characterizing the spatial transition relationships among landslide susceptibility types. To improve the readability of the transition matrix, we employed Sankey diagrams for visual analysis.

## 3. Results

### 3.1. Construction of Landslide Susceptibility Evaluation System

#### 3.1.1. Pearson Correlation Coefficient

**Figure 5:**
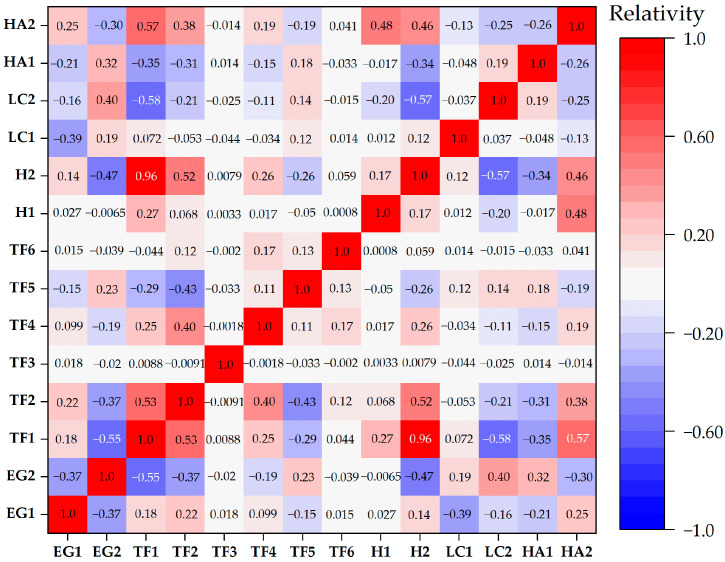
Pearson correlation coefficient matrix between assessment indicators.

Figure 5 shows the Pearson correlation coefficient (γ) between the 14 initial evaluation indicators. The correlation coefficient between TF1 and H2 is the highest (γ = 0.96), much higher than the threshold of 0.7, and the correlation coefficients between other factors are lower than 0.7. The strong correlation between TF1 and H2 is mainly because Yining County is located on the northern foothills of the Tianshan Mountains, characterized by complex and significantly undulating terrain, forming a stepped topographic structure. Moist westerly airflows advance from west to east and are forced to ascend due to the blockage of the Tianshan Mountains. As the air rises, it gradually cools, leading to water vapor condensation and the formation of abundant orographic rainfall. This process results in significantly higher annual precipitation in high-altitude areas compared to low-altitude plains and valleys. For every certain increase in elevation, precipitation shows a clear upward trend, leading to a high degree of consistency between the spatial distribution of precipitation and elevation changes at the macro scale. Simultaneously, the Beishan River system is extensively developed in the mountainous areas, forming a dense river network. These rivers primarily originate from high-altitude mountainous regions, with their runoff highly dependent on mountainous precipitation and seasonal snowmelt. Topographic uplift leads to concentrated rainfall, which in turn modifies surface and subsurface environments through hydrological processes. The chain relationship of “high altitude–abundant precipitation–strong runoff–high landslide susceptibility” is particularly prominent, further substantiating the comprehensive effect of high consistency between elevation and rainfall in the spatial distribution of Yining County.

There is a significant difference in spatial resolution between the two indicators: H2 has 1 km grid data, while TF1 has 30 m grid data that can better reflect topographic details. Therefore, this study excluded H2 as a redundant factor and retained TF1.

#### 3.1.2. Multicollinearity Test

**Figure 6:**
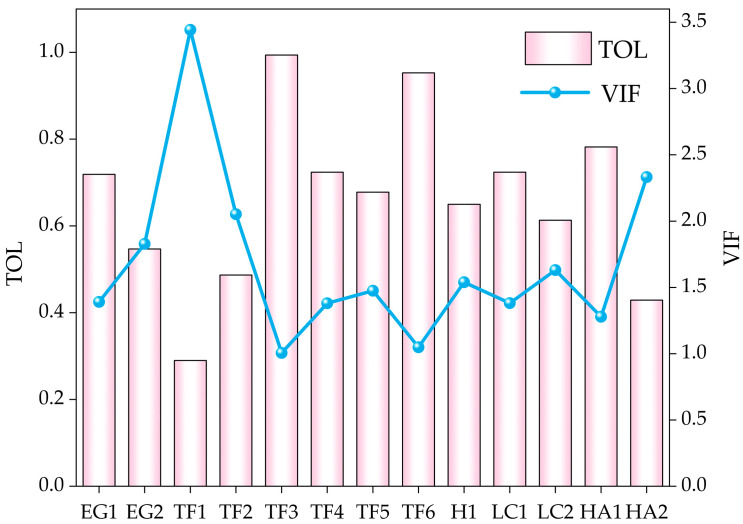
Collinearity diagnosis results of assessment indicators.

The 13 driving factors screened by the Pearson correlation coefficient were tested for multicollinearity, and the result is shown in Figure 6. The result shows that the VIFs of all evaluation factors are less than 10, and the TOLs are greater than 0.1, indicating that there is no significant multicollinearity among the 13 driving factors. Therefore, all factors after preliminary screening pass the collinearity test and can be input into the landslide susceptibility evaluation system.

#### 3.1.3. Importance Analysis of Evaluation Factors Based on RF

**Figure 7:**
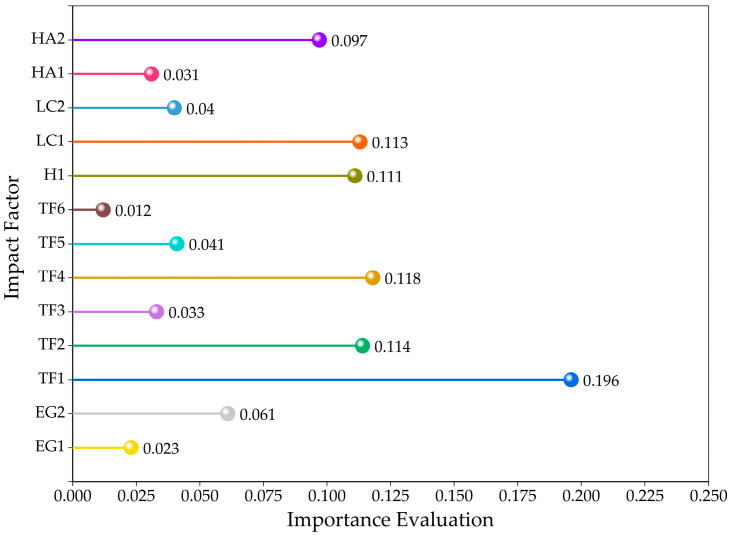
Evaluation factor importance score based on RF.

RF is used to analyze the Gini importance of landslide evaluation factors and calculate the importance score of each evaluation index. The result is shown in Figure 7. It can be seen that the importance score of TF1 is the highest, reaching 0.196, indicating that the altitude is the main factor inducing landslides, which is in line with the experience and understanding of landslide driving factors. With the increase in altitude, the rainfall in high-altitude areas is richer, vegetation is reduced, and landslides are more prone. The importance score of TF6 is less than 0.02, and its interpretation ability for landslides is weak. The importance scores of other factors are 0.02~0.12, indicating that these factors have different contributions to landslide occurrence. Therefore, TF6 is eliminated and the remaining factors are included in the landslide susceptibility evaluation system.

### 3.2. Discretization of Landslide Susceptibility Evaluation Factor

#### 3.2.1. Discretization of Continuity Factor

In this paper, we used the ChiMerge algorithm to discretize and grade continuous evaluation factors such as TF1, TF2, TF4, TF5, and LC1. The final division result is shown in Table 2. The discretization results of each factor and the distribution of landslides showed obvious laws: TF1 was divided into six sections, and landslides were mainly concentrated in the (1183, 1716) section at medium altitude. TF2 was divided into five sections, of which (23.41, 33.45] and (33.45, 71.08] sections had higher landslide density, indicating that steep slope areas are more prone to landslides. TF4 was divided into four sections, and landslide density was the largest in the (13.647, 2896.1] section, indicating that landslides were more likely to occur in areas with thick sediment accumulation. TF5 was divided into four sections, with a relatively high landslide density in the low-value interval [2.149, 4.315]. The low-value areas were mainly hillsides or steep terrain, where soil moisture was generally not high. This further indicates that landslides mainly occurred in areas with relatively large slopes. LC1 was divided into four sections, and landslide density was the largest in the interval (0.224, 0.583], which was consistent with the characteristics of poor slope stability in the area with sparse vegetation.

#### 3.2.2. Discretization of Linear Factor

The occurrence of landslides shows a law of continuous change with the distance from influencing factors such as rivers, roads, and faults. In areas close to rivers, roads, and faults, the frequency of landslides is significantly higher. As the distance increases, the frequency gradually decreases, showing a gradual change trend.

ChiMerge discretization was applied to segment the Euclidean distance of linear factors to determine the optimal distance segmentation point and influence range. The specific classification result is shown in Table 3. EG2 was divided into five categories, H1 was divided into three categories, and HA2 was divided into five categories. It was found that the landslide density decreased with the increase in EG2 and H1. The maximum influence distance of faults and rivers on landslides was 1026 m and 248 m. The relationship between HA2 and landslide density was “decrease–increase–decrease”. The highest landslide density occurred in the interval 1320~3543 m, which was mainly due to the uneven distribution of the road network in mountainous areas and plains.

### 3.3. Landslide Susceptibility Assessment Based on Machine Learning

#### 3.3.1. Landslide Susceptibility Assessment Results

**Figure 8:**
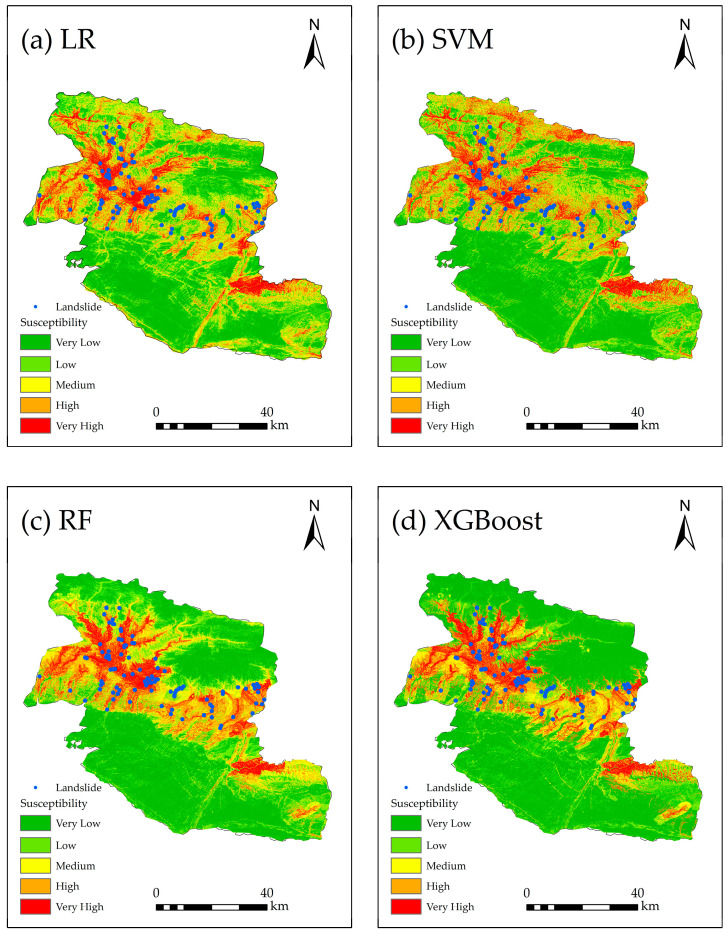
Landslide susceptibility zoning based on (**a**) LR, (**b**) SVM, (**c**) RF, and (**d**) XGBoost.

We used four machine learning models (LR, SVM, RF, and XGBoost) to conduct a landslide susceptibility assessment. The landslide susceptibility probability values of each model were divided into five levels—very high, high, medium, low, and very low—using the natural breaks method (Figure 8).

**Figure 9:**
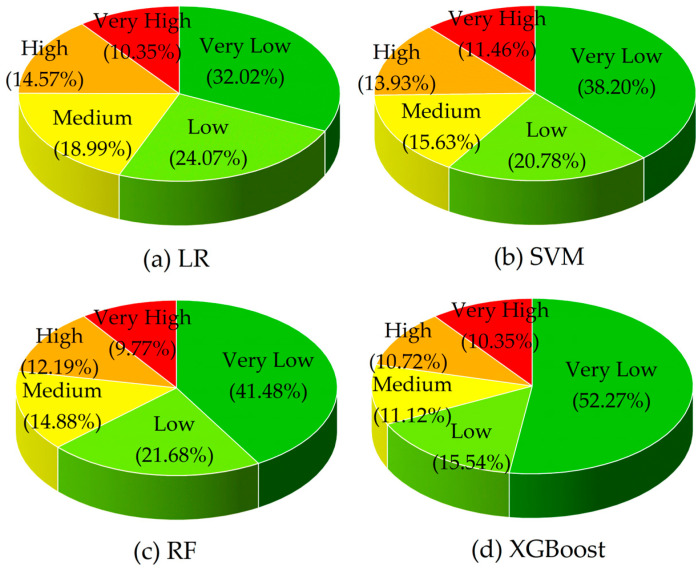
Statistics of landslide susceptibility zoning for each model.

In terms of the proportion of susceptibility levels in each model (Figure 9), the zoning results of the four models are generally consistent, which indicates that the spatial distribution of landslide susceptibility in this study area is similar, but there are significant differences in details. For the LR, the proportion of the very-high-susceptibility area is 10.35%, and approximately 46.39% of the area is classified as low or very low. For the SVM, the proportion of the very-high-susceptibility area is 11.46%, slightly larger than that of the LR, while the proportion of low- and very-low-susceptibility areas reaches 58.98%. For the RF, the proportion of the very-high-susceptibility area is 9.76%, the lowest among the four models, and the proportion of low- and very-low-susceptibility areas reaches 63.16%, the highest among the four models. For the XGBoost, the proportion of the very-high-susceptibility area is 10.35%, the same as that of the LR, and the proportion of the very-low-susceptibility area is the largest, reaching 52.27%. A comprehensive comparison shows that the RF identifies the smallest proportion of very-high-susceptibility areas, while the LR predicts a proportion of very-low-susceptibility areas, significantly smaller than the other three models, with a difference ranging from 6.18% to 20.25%. The high- and very-high-susceptibility areas predicted by the LR and SVM are relatively more scattered in spatial distribution, while the other two tree-based models (RF and XGBoost) show a more concentrated pattern.

We incorporated historical landslide data to further analyze the prediction performance of the four models. For the LR, SVM, RF, and XGBoost, the areas of high- and very-high-susceptibility zones are 1113.50 km^2^, 1134.27 km^2^, 980.84 km^2^, and 941.37 km^2^, respectively. Among these zones, the proportions of historical landslides account for 82.11%, 76.42%, 95.94%, and 91.46% of the total historical landslides. In the very-low-susceptibility zones, the proportions of historical landslides for the LR, SVM, RF, and XGBoost are 1.22%, 3.25%, 0%, and 1.22% respectively. Overall, the zoning results of the RF are more accurate and detailed. It has the highest proportion of historical landslides in the high- and very-high-susceptibility zones, making its prediction results the most reliable.

#### 3.3.2. Model Accuracy Evaluation

To comprehensively evaluate model performance, we used classification performance metrics and probability discrimination metrics to assess the predictive performance and results of the models. These metrics include Precision, Recall, F1-Score, Overall Accuracy (OA), and Kappa coefficient (Table 4). The results indicate that the Kappa coefficients of the RF and XGBoost exceed 0.6. This demonstrates that the RF and XGBoost have high reliability, reflecting the high sensitivity of tree-based models in capturing the nonlinear relationships associated with landslides. Furthermore, the RF achieves a relatively high OA (0.85) and Kappa coefficient (0.7003), which confirms that its performance advantage is not accidental. The LR has an OA value of 0.7566, but its Kappa coefficient is only 0.514. In conclusion, the models are ranked by performance from highest to lowest as RF > XGBoost > LR > SVM, with the RF exhibiting superior performance in landslide susceptibility assessment.

**Figure 10:**
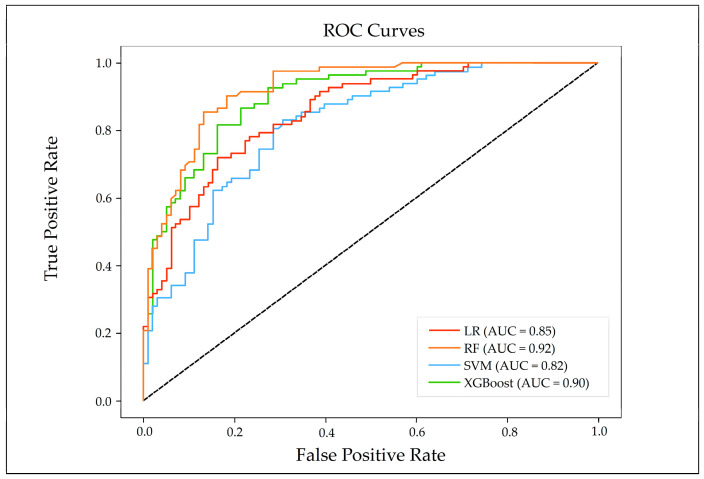
ROC curves of the four used models.

To further evaluate the predictive accuracy of the models, ROC curves are employed to analyze the prediction performance of the four models. When the AUC value is greater than 0.7, it indicates that the model has a good predictive effect. As shown in Figure 10, the AUC values of the LR, SVM, RF, and XGBoost are 0.85, 0.82, 0.92, and 0.90, respectively, all of which are higher than the baseline threshold of 0.7, indicating that the prediction effects of the four models are relatively good. Among them, the RF has the best prediction performance, followed by XGBoost, and the prediction performance of the LR is slightly better than that of SVM.

### 3.4. Landslide Susceptibility Assessment Incorporating SBAS-InSAR Deformation Characteristics

#### 3.4.1. SBAS-InSAR Land Deformation Results

**Figure 11:**
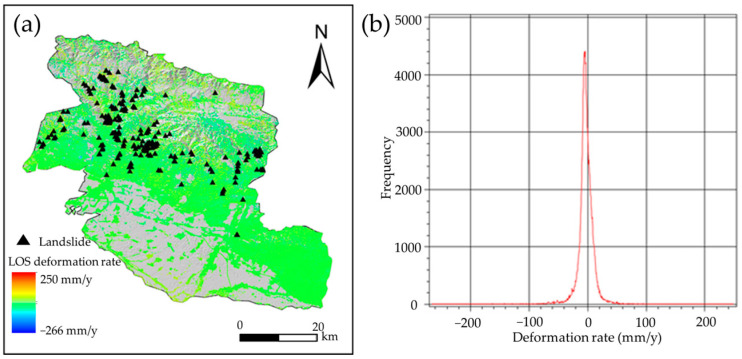
Land deformation in Yining County: (**a**) annual average deformation rate in Los direction; (**b**) distribution histogram of annual average deformation rate.

Based on the SBAS-InSAR technique, the land deformation during the monitoring period was obtained, as shown in Figure 11. In Yining County, the land deformation rate from 2022 to 2023 ranged from −266 to 250 mm/y; anomalous deformation mainly occurred along both sides of the valleys in the northern mountainous area and on the Aburele Mountain. The mean annual rate was −4 mm/y with a standard deviation of 13 mm/y, and the distribution was centered near 0, approximately following a normal distribution, indicating that the inversion results were reasonable and accurate.

#### 3.4.2. Landslide Susceptibility Assessment Combined with SBAS-InSAR Results

**Figure 12:**
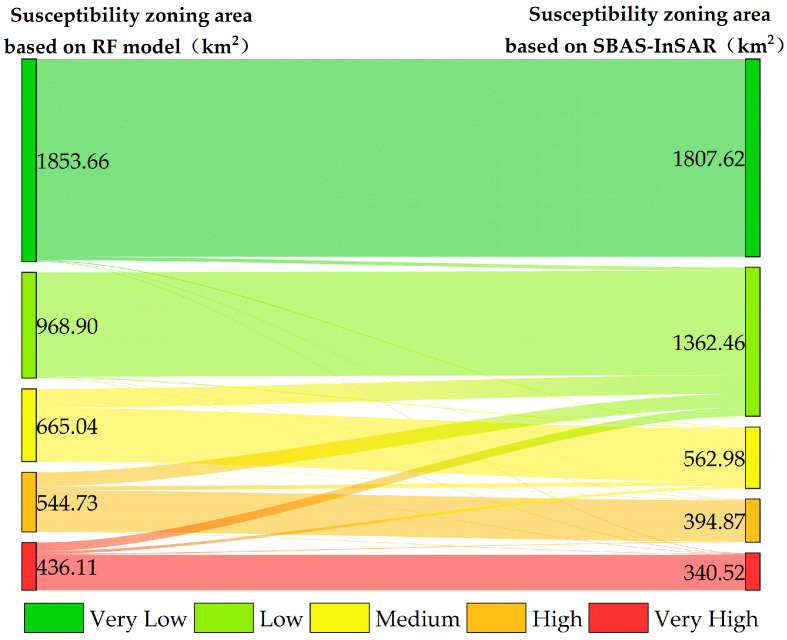
Statistical analysis of changes in landslide susceptibility zoning area based on RF and combined with SBAS-InSAR.

The land deformation information obtained based on the SBAS-InSAR technique could characterize the landslide deformation patterns within a specific time period. It introduced a near-real-time indicator of landslide activity status into susceptibility assessment, thereby improving the accuracy of landslide susceptibility assessment. Among the four aforementioned machine learning models, RF achieved the highest accuracy in its assessment results. Therefore, this study integrated SBAS-InSAR surface deformation data with the initial landslide susceptibility assessment results generated by the RF model. Using the statistical standard deviation of deformation rates as the classification criterion, a dynamic landslide susceptibility evaluation matrix (Table 5) was constructed. The application of this matrix optimized the generation process of the landslide susceptibility zoning map, thereby significantly enhancing the accuracy and reliability of the final landslide susceptibility zoning results. The changes in the zonal area between the initial RF susceptibility zoning and the optimized one (integrated with SBAS-InSAR results) are statistically analyzed, as illustrated in Figure 12.

Compared with the initial susceptibility results, the areas of the medium, high, and very-high zones decrease by 102.06 km^2^ (−15.4%), 149.87 km^2^ (−27.5%), and 95.59 km^2^ (−21.9%), respectively. The area of the low zone increases by 393.55 km^2^ (+40.6%), mainly being converted from the initial very-low and medium zones. For the RF assessment result, 23.4% of the high zone is converted to the medium zone, 25.9% of the medium zone is converted to the low zone, and 1.7% of the very-low zone is converted to higher-grade zones.

**Figure 13:**
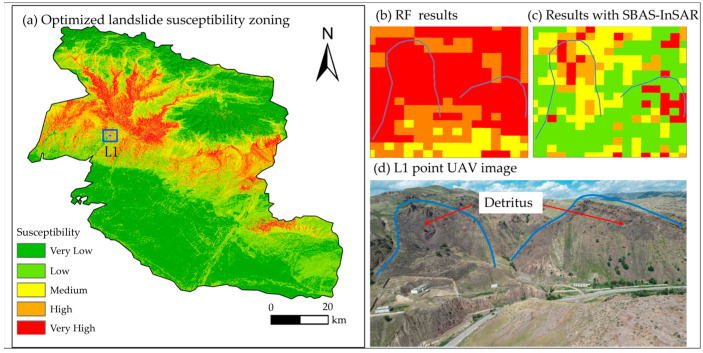
Landslide susceptibility zoning based on SBAS-InSAR deformation characteristics and comparison and field verification of different models.

To clearly highlight the differences between the landslide susceptibility maps before and after optimization, we selected point L1 (Figure 13a)—where the differences were relatively significant—for local magnification analysis (Figure 13b,c) and conducted verification analysis by integrating field survey data (Figure 13d). The results show that the optimized susceptibility zoning has higher accuracy in identifying landslide active areas: the red patches in Figure 13c (very high) accurately depict the sliding ranges of the two landslides at point L1. The slope in this area is relatively unstable, with obvious signs of fragmentation at the landslide rear edge and sliding traces of fragmented blocks. Compared with the results of the RF alone, the landslide susceptibility evaluation results integrated with SBAS-InSAR deformation results are more detailed and more consistent with the actual situation.

## 4. Discussion

### 4.1. Landslide Analysis Based on Administrative Divisions

High-quality landslide susceptibility zoning maps are crucial for the prevention and control of geological disasters and territorial spatial planning under the synergistic effects of mineral resource development and climate change. The spatial distribution characteristics of landslide susceptibility in Yining County are closely related to multiple factors such as topography and geomorphology, meteorology and hydrology, land cover, and human activities. Topographic and geomorphic types, faults, rock–soil mass types, and soil types jointly constitute the basic conditions for landslide gestation. Rivers affect the stability of slope feet. Human activities influence landslide occurrences by altering vegetation coverage and slope stress status. Furthermore, in the study area, precipitation shows a significant correlation with elevation, and rainfall often serves as a direct triggering factor for landslides (Table 6). The combined effects of natural conditions and human factors collectively control the spatial distribution pattern of landslide susceptibility in Yining County. In all regions of Yining County, both precipitation and landslide occurrence are most frequent during the summer (June–August). Additionally, persistent rainfall in autumn (September–October) may trigger a limited number of landslides, though their frequency and intensity are considerably lower than in summer. During the spring snowmelt period, small-scale landslides can occur, but they lack the support of concentrated rainfall processes.

The very-high- and high-susceptibility zones are mainly distributed in some grasslands within Karayagqi Township, Awulia Township, and Mazar Township. This area has seasonal rivers, with loess mid-mountains as the main landform type and well-developed geological structures. In addition, human activities such as road construction, mineral mining, and grazing are relatively frequent. The combined effects of internal and external conditions make this area highly prone to landslide disasters.

The medium-susceptibility zone generally surrounds the periphery of the high-susceptibility zone, and is mainly distributed in the piedmont hills and eroded areas of Karayagqi Township, Awulia Township, and Mazar Township. The soil types here are dominated by calcic soils and thin-layer soils, with a relatively large number of residential settlements that are scattered.

The low- and very-low-susceptibility zones are mainly distributed in the northern part of the county, the middle–high mountainous areas in the eastern part, and the plain areas in the southern part. The southern plain area is the main residential area for urban and rural residents and a concentrated area for production and daily life in the county. Its landform type is the piedmont alluvial–proluvial inclined plain and the Yili River alluvial plain, with a flat and open terrain. The land use is dominated by cropland and construction land, which basically do not have the conditions for landslides. The middle–high mountainous areas in the northern and eastern parts are mainly composed of hard massive lithologic granite, making landslides less likely to occur.

### 4.2. Analysis of Deformation Characteristics of Typical Historical Landslides

To further understand the deformation laws and characteristics of historical landslides, we selected two typical historical landslide clusters with large volumes that were adjacent to residential areas as the research objects. Based on the 2022~2023 land deformation data obtained by SBAS-InSAR technology, we conducted an in-depth analysis on activity status and deformation characteristics.

#### 4.2.1. Panjinbulake Group 6 Landslide Cluster

**Figure 14:**
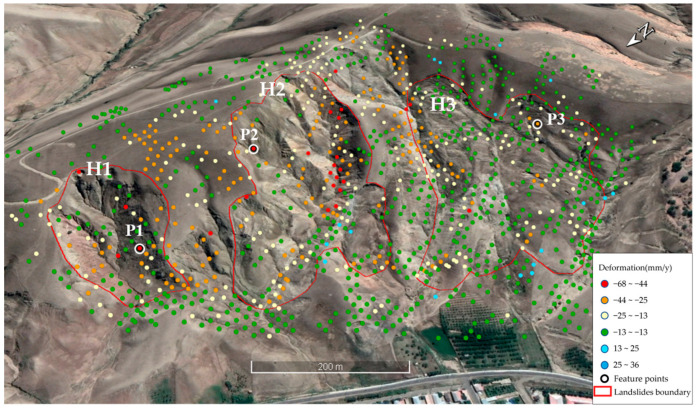
Deformation rate distribution map of the Panjinbulake Group 6 landslide cluster.

**Figure 15:**
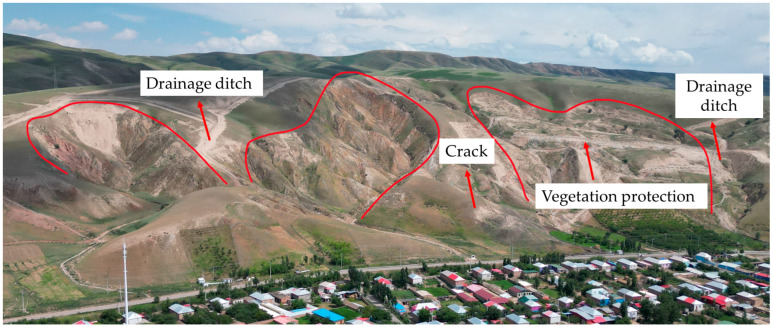
UAV imagery of the Panjinbulake Group 6 landslide group (July 2024).

The Panjinbulak Group 6 landslide cluster is located in Karayagaqi Township, adjacent to residential areas. It is a loess landslide with well-developed cracks. This landslide cluster exhibits an overall zonal distribution, with a length of approximately 800 m and a width of about 470 m, consisting of three potential landslide bodies (Figure 14). The land deformation rates in the landslide cluster area range from −68 mm/y to 36 mm/y, with an average deformation rate of −15 mm/y. Among them, the maximum deformation rates of H0, H1, and H2 are −51 mm/y, −66 mm/y, and −38 mm/y, respectively. Specifically, the front edges of the H0 and H1 landslides are relatively stable, but obvious deformation occurs at the sidewalls of the landslide masses and local rear edges of the landslides. The H2 landslide shows minimal overall deformation and is in a relatively stable state. Slope control projects have been implemented for this landslide cluster: drainage ditches have been constructed in the trenches on both sides for water drainage and diversion, and vegetation belts have been laid in the sliding surface area to enhance stability (Figure 15).

**Figure 16:**
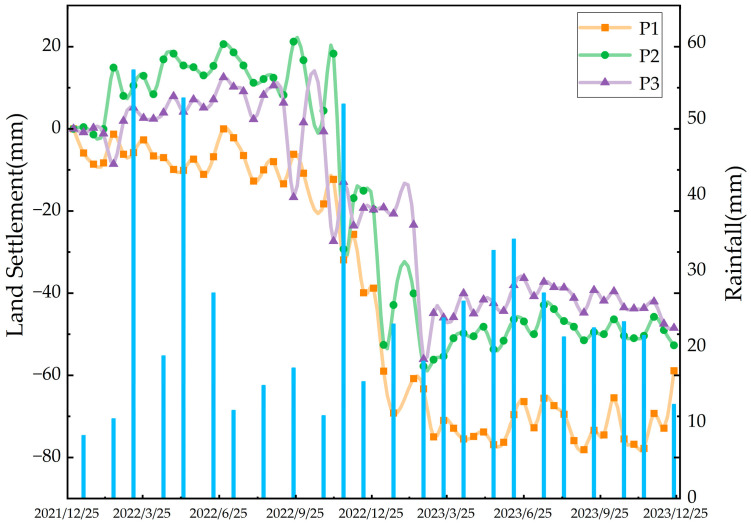
Time-series displacement curves of typical feature points and precipitation variations in the Panjinbulake Group 6 landslide cluster.

Combined with monthly precipitation data, the temporal deformation characteristics of feature points P1, P2, and P3 were analyzed (Figure 16). Overall, the deformation entered an accelerated deformation stage after November 2022. The maximum deformation amounts of feature points P1, P2, and P3 reached −58 mm, −78 mm, and −56 mm, respectively. The deformation of the feature points showed an unstable trend during the rainy season (May~August), reflecting the dynamic impact of rainwater infiltration on slope stability. The rainfall in November 2022 was higher than the average for the same period in normal years; affected by this, the deformation curves of the landslide feature points showed a significant downward trend after November 2022. Specifically, before November 2022, the cumulative deformation of the feature points was relatively stable, maintaining at −18~0 mm (P1), −1~21 mm (P2), and −17~10 mm (P3), respectively. From November 2022 to March 2023, the cumulative deformation of the feature points increased rapidly, reaching −75~−32 mm (P1), −57~−15 mm (P2), and −56~−13 mm (P3), respectively, entering the accelerated deformation stage.

#### 4.2.2. Landslide Cluster North of Qinghua Coal Mine in Karayagqi Township

**Figure 17:**
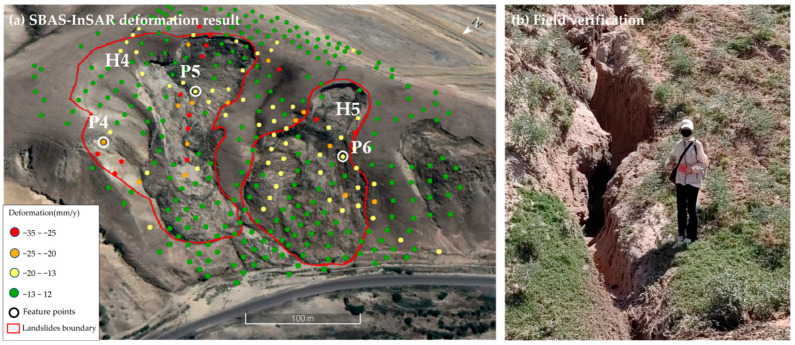
SBAS-InSAR deformation monitoring results and field verification of landslide cluster north of Qinghua Coal Mine in Karayagqi Township.

The landslide cluster north of Qinghua Coal Mine in Karayagaqi Township is adjacent to County Road 700. This landslide cluster has a length of approximately 240 m and a width of about 340 m, consisting of two landslide bodies (Figure 17a). From 2022 to 2023, the deformation rate of this landslide cluster ranged from −35 mm/y to 12 mm/y, with an average deformation rate of approximately −10 mm/y and a maximum cumulative deformation of −35 mm. Severe deformation occurred at the rear edge and the middle part of the landslide cluster; among them, the maximum deformation rates of the H3 and H4 landslides were −35 mm/y and −29 mm/y, respectively. The surface of the landslide is covered with loose loess, and deep longitudinal cracks have developed on both sides of the slope (Figure 17b), which makes the slope prone to instability induced by freeze–thaw cycles and rainfall.

**Figure 18:**
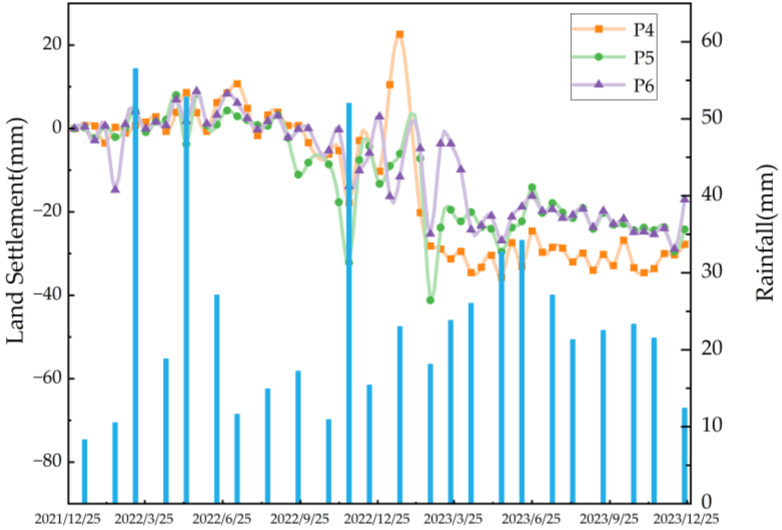
Time-series displacement curves of typical feature points and precipitation variations in landslide cluster north of Qinghua Coal Mine in Karayagqi Township.

In the sidewall and rear-edge areas of the H3 and H4 landslides where deformation is concentrated, feature points P4, P5, and P6 are selected for temporal deformation analysis, and the changes in displacement are shown in Figure 18. The results indicate that this landslide cluster is in a slow active stage. By analyzing the spatiotemporal evolution and deformation characteristics of the feature points in combination with precipitation data, it can be seen that in months with relatively high precipitation, all three feature points experience different degrees of settlement, and the deformation rate shows an increasing trend. The precipitation form from January to February is mainly sleet and snow, with a cumulative precipitation of 51 mm. In middle- and late-February, affected by snowmelt, the surface soil becomes saturated and softened, and its erosion resistance decreases sharply; meanwhile, frozen soil stagnant water and repeated freeze–thaw cycles cause dual damage. The superposition of precipitation infiltration and diurnal temperature difference further lead to slope stress imbalance and deformation. Therefore, the combined effect of meltwater infiltration and freeze–thaw cycles is the core factor inducing the frequent occurrence of geological hazards during this period. In addition, from May to June 2023, the regional precipitation is relatively large and concentrated, and the deformation rate of the landslide feature points accelerates significantly. The average deformation amounts of P4, P5, and P6 reach −30 mm, −23 mm, and −20 mm, respectively; after that, the feature points continue to deform with a small amplitude. Overall, freeze–thaw cycles and rainfall are the main inducing factors for the movement of this landslide. The infiltration of meltwater and rainwater leads to soil saturation and increased gravity, which promote slope deformation.

### 4.3. Coupling Mechanism Between Machine Learning and SBAS-InSAR

To address the limitations of traditional GIS-based landslide susceptibility assessment methods, which rely on expert weighting and implicit linear assumptions leading to subjective uncertainty, this study proposes a dynamic optimization framework that integrates machine learning with SBAS-InSAR deformation monitoring. Traditional methods often suffer from evaluation biases due to spatial heterogeneity in complex mountainous areas, while machine learning models capture nonlinear relationships between multi-source environmental factors and landslide occurrences. In the landslide susceptibility assessment of Yining County, the Random Forest (RF) model demonstrated high predictive accuracy (AUC value of 0.92), validating its applicability under complex geological conditions.

However, existing machine learning studies primarily focus on static indicators such as topography and geology, with insufficient characterization of the dynamic deformation features of landslides. To address this, this study constructs a coupling mechanism between machine learning and SBAS-InSAR: the former serves as the base model for static susceptibility zoning, while the latter provides time-series deformation information for dynamic correction. The core of this mechanism lies in the design of a post-processing dynamic correction strategy—instead of retraining the model using deformation rates as features, it spatially reallocates the weights of the initial zoning results by constructing a “susceptibility–deformation” correlation matrix. This approach avoids model instability caused by spatiotemporal resolution inconsistencies in InSAR data while preserving the structural stability of the Random Forest model.

The coupling mechanism is implemented through a three-tier process: first, initial susceptibility classification is generated based on the Random Forest model; second, dynamic activity levels are delineated using the standard deviation of SBAS-InSAR deformation rates; and finally, spatial coupling between the two is achieved through an optimized evaluation matrix, producing a dynamic information-integrated susceptibility zoning map.

This “static modeling + dynamic correction” coupling paradigm not only enhances the practical consistency and spatiotemporal adaptability of landslide susceptibility assessment but also provides an extensible technical pathway for landslide risk assessment in data-scarce or rapidly changing surface environments. Future work could involve introducing deep learning methods to enhance automatic feature extraction and combining multi-source time-series remote-sensing data to further optimize dynamic correction accuracy, advancing landslide risk assessment toward intelligent and real-time development.

## 5. Conclusions

Taking Yining County, Xinjiang, as the study area, this study constructed a landslide susceptibility evaluation system from five aspects: engineering geology, topography and feature, hydrometeor, land cover, and human activity. Four machine learning-based landslide susceptibility prediction models were developed and the optimal prediction model suitable for the characteristics of the study area was selected. Based on the SBAS-InSAR technique, land deformation features were extracted. The land deformation results were combined with the optimized susceptibility prediction model to conduct regional landslide susceptibility evaluation, and the application effect and reliability of this integrated approach in landslide susceptibility mapping were explored. The main conclusions are as follows:

(1) Through Pearson correlation analysis, a multicollinearity test, and RF importance ranking, the rainfall index—with a correlation coefficient of 0.96 with elevation—and the profile curvature index—with an importance score of less than 0.02—were excluded. Finally, 12 indicators were selected, and the ChiMerge method was used to discretize the continuous indicators, thereby constructing the landslide susceptibility evaluation system.

(2) Four machine learning methods (LR, SVM, RF, and XGBoost) were applied to conduct landslide susceptibility evaluation. The overall characteristics of the results were relatively consistent, but there were significant differences in specific details. The model performance was ranked as RF (0.92) > XGBoost (0.90) > LR (0.85) > SVM (0.82), indicating that the RF was the most suitable susceptibility prediction model for Yining County.

(3) The monitoring results of the SBAS-InSAR technique show that the land deformation rate in Yining County ranged from −266 mm/y to 250 mm/y. By integrating the deformation results with the RF, an optimized landslide susceptibility zoning result was generated, which realized the refined assessment of landslide susceptibility zoning.

This study can accurately delineate landslide-prone areas and optimize disaster prevention and mitigation arrangements. While safeguarding the lives and property of the people, it provides solid scientific support for the regional planning and sustainable development of Yining County and similar mountainous cities. In the future, we will further incorporate data on seismic activity, energy and mineral development intensity, and other relevant factors to quantitatively analyze their impacts on landslide occurrences in Yining County, thereby improving the landslide susceptibility evaluation system. Meanwhile, we will deeply integrate InSAR technology, deep learning methods, high-resolution optical images, LiDAR data to enhance the dynamic monitoring capability for sudden landslides.

## Figures and Tables

**Table 1:** Relevant information on landslide driving factor data.

Data Type	Data	Data Source	Resolution (m)	Abbreviation
Engineering Geology	Lithology	https://www.ngac.cn/125cms/c/qggnew/index.htm (accessed on 20 July 2025)	30	EG1
	Distance from Fault		30	EG2
Topographic Features	DEM	https://www.gscloud.cn/home (accessed on 20 July 2025)	30	TF1
	Slope		30	TF2
	Aspect		30	TF3
	Sediment Transport Index		30	TF4
	Topographic Wetness Index		30	TF5
	Profile Curvature		30	TF6
Hydrometeor	Distance from River	https://www.openstreetmap.org (accessed on 20 July 2025)	30	H1
	Annual Average Precipitation	https://nnu.geodata.cn/index.html (accessed on 20 July 2025)	1000	H2
Land Cover	NDVI	GEE	10	LC1
	Soil Type	https://www.fao.org (accessed on 1 September 2025)	30	LC2
Human Activity	Land Use	https://www.esa.int/ (accessed on 1 September 2025)	10	HA1
	Distance from Road	https://www.openstreetmap.org (accessed on 1 September 2025)	30	HA2

**Table 2:** Discretization results of continuous factors.

Continuity Factor	$\chi^{2}$	Freedom Degree	Interval Division Results	Section Area (km^2^)	Landslide Density (Piece/km^2^)
TF1	235.9	5	596–908	1506.89	0.01
			908–1183	796.17	0.06
			1183–1716	897.79	0.19
			1716–1964	403.06	0.04
			1964–2493	639.74	0.002
			2493–3594	224.84	0
TF2	123.869	4	0–7.53	1590.59	0.01
			7.53–14.32	1164.89	0.039
			14.32–23.41	865.73	0.07
			23.41–33.45	551.61	0.14
			33.45–71.08	285.05	0.17
TF4	126.717	3	0–2.424	1408.91	0.01
			2.424–7.682	1295.86	0.03
			7.682–13.647	712.48	0.08
			13.647–2896.1	1040.62	0.13
TF5	36.64	3	2.149–4.315	595.63	0.13
			4.315–5.354	1206.92	0.06
			5.354–8.505	1946.53	0.04
			8.505–28.63	708.79	0.03
LC1	121.376	3	−0.6–0.224	851.89	0.07
			0.224–0.583	1341.12	0.11
			0.583–0.779	887.98	0.04
			0.779–1	1386.32	0.01

**Table 3:** Discretization results of linear factors.

Continuity Factor	$\chi^{2}$	Freedom Degree	Interval Division Results	Section Area (km^2^)	Landslide Density (Piece/km^2^)
EG2	60.605	4	0–1026	910.44	0.09
			1026–2037	680.33	0.07
			2037–2778	383.01	0.06
			2778–9652	1594.20	0.05
			9652–33,023	900.52	0
H1	77.556	3	0–248	425.94	0.19
			248–1429	1654.68	0.05
			1429–3730	1616.51	0.04
			3730–9609	771.35	0.01
HA2	83.337	4	0–553	1181.39	0.06
			553–1320	887.91	0.04
			1320–3543	1178.45	0.08
			3543–6115	546.82	0.06
			6115–16,192	673.91	0.01

**Table 4:** Accuracy of models versus the test set.

Model	Precision (%)	Recall (%)	F1-Score (%)	OA (%)	Kappa (%)
LR	76.52	75.56	75.58	75.66	51.40
SVM	75.72	72.22	71.86	72.22	45.63
RF	85.48	85.00	85.03	85	70.03
XGBoost	82.34	81.67	81.70	81.67	63.44

**Table 5:** Landslide susceptibility optimization assessment matrix.

Landslide Susceptibility	Surface Deformation Rate (mm/y)				
	[−13, 13)	[−25, −13)⋃[13, 25)	[−35, −13)⋃[25, 35)	[−45, −35)⋃[35, 45)	[−266, −45)⋃[45, 250)
Very Low	0	+1	+2	+3	+4
Low	0	0	+1	+2	+3
Medium	−1	0	0	+1	+2
High	−2	−1	0	0	+1
Very High	−3	−2	−1	0	0

**Table 6:** Statistics of precipitation and landslide susceptibility area by township.

Township	Precipitation/(mm/y)	Proportion of Susceptibility Zone Types by Area/(%)				
		Very High	High	Medium	Low	Very Low
Jiliyuzi Town	312.71	5.18	5.87	12.16	30.86	45.93
Dunmazha Town	266.94	0.11	0.50	6.05	40.41	52.93
Hudiyayuzi Town	348.70	2.34	2.25	5.94	21.04	68.43
Tulufanyuzi Township	321.48	2.54	4.26	10.07	32.60	50.53
Kalayagqi Township	369.82	20.12	18.03	20.06	26.91	14.88
Yuqunweng Hui Ethnic Township	310.455	2.28	5.18	8.92	19.36	64.26
Wugong Township	327.65	1.65	3.92	9.72	26.31	58.38
Yingtamu Town	283.12	1.66	2.95	5.21	13.22	76.97
Bayituohai Town	271.68	0.10	0.59	3.27	19.52	76.53
Uygur Yüqiwen Town	256.06	0.05	0.54	5.20	28.27	65.92
Samuyuzi Town	282.28	1.08	1.80	4.78	38.52	53.83
Kashi Town	283.31	2.24	2.24	5.45	43.07	46.99
Mazha Township	345.89	8.30	11.61	16.02	38.11	25.97
Wenyar Town	296.13	0.83	0.85	4.34	25.61	68.38
Awuliya Township	355.36	5.45	12.26	18.41	33.21	30.67
Quluhai Township	307.25	2.30	6.20	15.77	36.61	39.11
Arewusitang Town	294.49	0.31	1.23	4.88	22.65	70.93
Sadikeyuzi Township	317.98	0.66	0.97	3.34	20.76	74.27

## Data Availability

The data presented in the study are available on request from the first and corresponding author.
